# Fabrication of silk fibroin peptide–astaxanthin nanocomposites by nanoprecipitation for enhanced stability and antioxidant activity

**DOI:** 10.3389/fnut.2026.1803229

**Published:** 2026-07-07

**Authors:** Stopira Yannick Benz Boboua, ShaoPeng Chen, Yong Sun, Mengjie Li, Ying Guo, QingMei Wen, Yilu Chen, Zeng Fan, Tao Zheng

**Affiliations:** 1 College of Engineering, Northeast Agriculture University, Harbin, China; 2 Guangzhou Institute of Energy Conversion, Chinese Academy of Sciences, Guangzhou, China; 3 College of Grassland Science and Technology, China Agricultural University, Beijing, China; 4 Ecogreen Biotechnology Research Institute, Nanjing, China; 5 School of Environmental Engineering, Nanjing Institute of Technology, Nanjing, Jiangsu, China

**Keywords:** antioxidant stability, astaxanthin, nanocomposite, nanoprecipitation, silk fibroin peptide

## Abstract

Astaxanthin (AST), a potent antioxidant, suffers from poor stability and low solubility, limiting its applications across various fields. To address these issues, this study aims to develop a silk fibroin peptide-astaxanthin (SFP-AST) nanocomposite using a simple nanoprecipitation method. Characterization techniques such as Ultraviolet–visible spectroscopy (UV-vis), fluorescence spectroscopy, circular dichroism (CD), transmission electron microscopy (TEM), dynamic light scattering (DLS) and Fourier Transform Infrared (FTIR) confirmed that SFP stabilizes AST in nanoparticles ranging in size from 150 to 300 nm. Stability testing showed that under alkaline shock conditions, the retention rate of AST in the nanocomposite was 55.6%, whereas the retention rate in the free AST solution was only 43.6%. After 24 h of storage, the retention rate of AST in the nanocomposite was 44.4%, whereas the retention rate in the free AST solution was only 30.8%. Furthermore, 2,2-diphenyl-1-picrylhydrazyl (DPPH) radical scavenging assays revealed that the SFP-AST nanocomposite exhibited a 62.40% radical scavenging activity, compared to 38.11% for free AST. These results suggest that silk fibroin peptides can effectively stabilize and enhance AST, offering a natural, biocompatible carrier with broad potential applications in functional foods, dietary supplements, and cosmetics.

## 1 Introduction

Astaxanthin (AST) is a carotenoid known for its potent antioxidant activity and broad health benefits. Structurally, AST comprises a polyene chain with keto groups on either side, attached to β-ionone rings. The hydroxyl group within the ring structure, together with the hydroxyl (OH) and carbonyl (C=O) functional groups, contributes to its antioxidant properties. The presence of oxygen in the ionone rings confers a polar character to astaxanthin (1, 2). Its principal mechanism for scavenging free radicals involves electron arrangement, enabling it to donate an electron readily to neutralize unstable reactive oxygen species. The hydroxyl groups in the rings can form esters with fatty acids, resulting in monoesters and diesters with one or two fatty acids, respectively. Free forms of astaxanthin are susceptible to oxidation. Therefore, naturally occurring astaxanthins are either conjugated with proteins or esterified with one or two fatty acids to form stable compounds (3–5). In the food industry, AST is used as a natural pigment for coloring, and as a preservative to extend the shelf life of foods and enhance their nutritional value. In the pharmaceutical field, AST’s antioxidant and anti-inflammatory properties have shown potential in the prevention and treatment of various diseases, including cardiovascular ailments, cancers, and neurodegenerative disorders (6, 7). The cosmetics industry has also capitalized on AST’s antioxidant and photo-protective effects in anti-aging and sunscreen products. Additionally, astaxanthin is widely used in aquaculture to improve the immunity and growth of aquatic animals (8, 9). These diverse applications underscore astaxanthin’s importance, but they also demand effective strategies to maintain its stability and efficacy in different environments.

Despite its benefits, astaxanthin is chemically unstable and prone to degradation under environmental stressors such as light, heat, pH extremes, and metal ions. Light exposure, for example, accelerates AST degradation, while high temperatures and strongly acidic or basic conditions significantly reduce its stability. Certain metal ions (e.g., Fe^3+^, Cu^2+^) can also catalyze the degradation of astaxanthin (5, 10–12). Furthermore, AST has very low water solubility and tends to degrade during processing and storage, which together limit its bioavailability and practical use in food, medical, and cosmetic products. These challenges necessitate the development of stabilization techniques to protect astaxanthin’s functional properties during formulation and usage.

To address the instability of astaxanthin, researchers have explored a variety of stabilization and delivery systems. Encapsulation of AST in carriers such as emulsions, microcapsules, liposomes, and polymeric nanoparticles has been shown to improve its water dispersibility and chemical stability, thereby enhancing its bioavailability (13–15). For instance, nano-emulsion and microencapsulation techniques can significantly extend AST’s shelf life by leveraging the protective effects of wall materials and the small size of the encapsulated particles. Likewise, liposomal and nanoparticle-based delivery systems improve the stability and controlled release of astaxanthin, taking advantage of their unique physical properties and biocompatibility (16, 17). The advancement of these encapsulation technologies has greatly expanded astaxanthin’s potential applications across various fields (18).

In recent years, bioactive peptides derived from protein hydrolysates have attracted widespread attention due to their diverse functional properties, including antioxidant, antihypertensive, antibacterial, and metabolic regulatory effects. These peptides not only exhibit significant biological activity but also demonstrate good biocompatibility and structural controllability. For example, studies have shown that hydrolyzed collagen from various sources possesses significant biological activity and holds broad application potential in the food and biomedical fields (19). Similarly, active peptides derived from casein have demonstrated anti-diabetic potential comparable to metformin in *in vitro* and gene expression analyses, highlighting their significant role in health promotion (20). Furthermore, the polypeptide profiles formed in fermented foods (such as camel milk cheese) have been shown to significantly influence their functional properties and bioactivity (21).

Silk fibroin protein, derived from silkworm cocoons, is a natural protein known for its excellent mechanical properties, biocompatibility, and biodegradability (18, 22, 23). In particular, enzymatically hydrolyzed silk fibroin peptide (SFP) has attracted attention as a functional biomaterial due to its unique amphiphilic properties and ability to self-assemble (24). Under appropriate conditions, SFP molecules can form nanostructures such as nanotubes or spherical nanoparticles, and they have been explored as carriers for drugs and bioactive compounds (25). For example, silk fibroin-based coatings have been used to preserve perishable foods by forming an edible barrier that slows down respiration and moisture loss, thereby extending shelf life (26). These attributes suggest that SFP could serve as a promising stabilizer and delivery matrix for sensitive bioactives like astaxanthin (27).

Nanoprecipitation is a facile and efficient method for preparing nanoparticles, offering several advantages including simplicity, speed, scalability, and a high degree of control over particle characteristics (28–30). In a typical nanoprecipitation process, an organic solution of the hydrophobic active compound is rapidly mixed with an anti-solvent (usually water), leading to the instantaneous formation of nanoscale precipitates (27). The resulting nanoparticle size, morphology, and surface properties can be finely tuned by adjusting parameters such as the solvent-to-anti-solvent ratio, mixing rate, and temperature (31–33). Nanoparticle encapsulation via nanoprecipitation has been demonstrated to improve the stability of various drugs and nutraceuticals. Surface modifications or co-assembly techniques can further impart targeting capabilities, thereby increasing the concentration of active compounds in specific tissues or cells and ultimately improving their bioavailability and therapeutic efficacy (34).

In this work, we propose a nanoprecipitation-based strategy to construct a silk fibroin peptide–astaxanthin (SFP-AST) nanocomposite that enhances the physicochemical stability and antioxidant activity of astaxanthin. SFP and AST are co-assembled by a rapid mixing/evaporation process to form SFP-AST nanoparticles. We thoroughly characterize the formation and properties of these nanocomposites using UV-visible spectroscopy, fluorescence spectroscopy, circular dichroism (CD), transmission electron microscopy (TEM), and dynamic light scattering (DLS). These analyses elucidate the binding mechanism between SFP and AST. We further evaluate the stability of AST in the SFP-AST nanocomposites under harsh conditions (exposure to alkaline pH and prolonged storage) and assess the antioxidant capacity of the composite using a DPPH free radical scavenging assay. Our results demonstrate that SFP can effectively stabilize astaxanthin and maintain its bioactivity, highlighting a promising approach to broaden the application of astaxanthin in functional foods, pharmaceuticals, and cosmetics (35).

## 2 Materials and methods

### 2.1 Materials

Silk fibroin peptide (SFP; molecular weight <500 Da) was purchased from Hangzhou Linran Biotechnology Co., Ltd. (Hangzhou, China). Astaxanthin (AST; C_40_H_52_O_4_, molecular weight 596.84) was obtained from Henan Putian Tongchuang Biotechnology Co., Ltd. (Henan, China). Sodium hydroxide (NaOH), hydrochloric acid (HCl), tetrahydrofuran (THF), and absolute ethanol were purchased from Sinopharm Chemical Reagent Co., Ltd. 2,2-Diphenyl-1-picrylhydrazyl (DPPH) and copper (II) chloride dihydrate (CuCl_2_·2H_2_O) were obtained from Aladdin Reagent Inc. All chemicals were of analytical grade or higher and used as received. Deionized water (DI water) was used for all preparations.

### 2.2 Sample preparation

A stock solution of SFP (10 mg/mL) was prepared using deionized water. A series of astaxanthin solutions in tetrahydrofuran (THF) was prepared as follows: Different amounts of astaxanthin were dissolved in 1 mL of THF to prepare AST-THF solutions with concentrations ranging from 0.001 mg/mL, 0.002 mg/mL, 0.004 mg/mL, 0.006 mg/mL, 0.008 mg/mL, and 0.01 mg/mL. Afterward, to prepare the SFP-AST nanocomposite, 1 mL of AST-THF solution (concentration adjusted as needed) was added dropwise to the SFP stock solution at a rate of 1 mL/min while stirring at 200 rpm. The organic solvent (THF) rapidly diffused into the aqueous phase, causing AST to precipitate and combine with SFP. After mixing, the mixture solution was stirred at 300 rpm at 25 °C for 3 h to ensure complete mixing. The solution was then incubated at 45 °C for 6 h to allow partial evaporation, followed by standing at room temperature overnight. This yielded a THF-free SFP-AST solution; the resulting SFP-AST nanoparticle suspension was immediately analyzed or subjected to further processing as described below. For comparison, a control sample of free AST was prepared in parallel (under the same solvent conditions but without SFP) and processed.

### 2.3 Ultraviolet–visible spectrophotometry (UV-vis)

The UV-vis absorption spectra of free astaxanthin and SFP-AST nanocomposites were recorded using a Shimadzu UV-2550 UV-vis spectrophotometer (Shimadzu, Japan). Samples were measured in quartz cuvettes over the wavelength range of 350–600 nm. Baseline correction was performed using the appropriate solvent blank. The characteristic AST absorbance (around 470 nm) was monitored to evaluate the formation of nanocomposites and the retention of AST in stability tests (22).

Encapsulation efficiency is calculated based on the difference between the total added AST and the residual free (unencapsulated) AST in the supernatant after nanoparticle formation, using ultraviolet–visible absorption at 470 nm (or the peak wavelength after red shift). The calculation formula is as follows:


$$\mathrm{EE}(\%)=\frac{C_{\text{Control}}V-C_{\text{sample}}V}{C_{\text{total}}V}$$

where $C_{\text{total}}$ is the initial AST concentration and $C_{\text{Control}}$ is AST concentration in the experimental group with water and free astaxanthin (W-AST) group and $C_{\text{sample}}$ is free AST concentration in the SFP-AST group determined from calibration curves of AST standards. V represents the volume of each sample, 1 mL. All measurements were performed in triplicate (*n* = 3) and the mean ± standard deviation is reported.

### 2.4 Characterization of SFP-AST nanocomposites

#### 2.4.1 Transmission electron microscopy (TEM)

The morphology of SFP alone and SFP-AST nanocomposites was observed by transmission electron microscopy (Hitachi 7,800, Japan). An aliquot of each sample (30 μL) was deposited onto a 400-mesh carbon-coated copper grid (Canemco-Marivac, Canada) and allowed to adsorb for ~5 min. The grid was then gently blotted to remove excess liquid and stained with 2% (w/v) phosphotungstic acid solution (10 μL) for 30 s. Excess stain was wicked off with filter paper, and the grid was rinsed with a drop of DI water to remove residual stain. After air-drying, the samples were examined using a Hitachi HT7800 TEM (Hitachi, Japan) at an accelerating voltage of 120 kV. Digital images were captured to assess particle size and morphology (18).

#### 2.4.2 Dynamic light scattering (DLS) and zeta potential

The hydrodynamic diameter and zeta potential of the nanocomposites were measured on a Malvern Zetasizer Nano ZS90 system (Malvern Instruments, United Kingdom). For particle size measurements, 50 μL of the SFP-AST sample was diluted in DI water and injected into a disposable plastic microcuvette. Measurements were taken at 25 °C after an equilibration time of 120 s, and each sample was measured in triplicate. The average hydrodynamic diameter (*z*-average) and polydispersity index were recorded. To monitor size stability, the SFP-AST sample was stored at ambient conditions and its hydrodynamic diameter was re-measured over a period of 5 days. Zeta potential was determined by diluting 50 μL of sample into 1 mL of DI water and loading it into a DTS1070 folded capillary cell. The electrophoretic mobility was measured and converted to zeta potential using the Smoluchowski model. The refractive index of the dispersant (water) was set to 1.330, and measurements were performed at a fixed scattering angle of 175 ° (backscattering mode). All DLS and zeta potential data are reported as mean ± standard deviation from three measurements (36).

#### 2.4.3 Fourier transform infrared (FTIR) spectroscopy

FTIR spectra were collected to analyze the secondary structure of SFP and any structural changes upon AST binding. A Nicolet iS20 FTIR spectrometer (Thermo Fisher Scientific, United States) equipped with an ATR (attenuated total reflectance) accessory was used. The spectrometer was allowed to warm up for 15 min prior to analysis. For each measurement, a drop of liquid sample was placed onto the ATR crystal (diamond/ZnSe), ensuring full coverage of the crystal surface without bubbles. A background spectrum (air) was first recorded under the same conditions, then the sample spectrum was acquired. Spectra were collected in the range of 4,000–400 cm^−1^ with a resolution of 4 cm^−1^ and 32 scans were averaged per spectrum. After each run, the ATR crystal was thoroughly cleaned with absolute ethanol and dried. All spectra were baseline-corrected, and key peaks (especially in the amide I region around 1600–1700 cm^−1^) were analyzed to assess the protein secondary structure (18).

#### 2.4.4 Circular dichroism (CD) spectroscopy

Circular dichroism measurements were performed to further examine the secondary structure of SFP before and after forming nanocomposites with AST. Far-UV CD spectra were recorded on a JASCO PTC-348 W1 spectropolarimeter (JASCO Inc., Japan). SFP solution (1 mg/mL in water) and SFP-AST nanocomposite solution (containing 1 mg/mL SFP and 0.01 mg/mL AST) were loaded into a 1 mm pathlength quartz CD cuvette (300 μL sample volume). Spectra were scanned from 175 nm to 250 nm at 25 °C with a scanning speed of 50 nm/min and a bandwidth of 1 nm. Each spectrum represents the average of three scans and was corrected by subtracting the corresponding solvent baseline. The CD data (molar ellipticity) were analyzed to detect any changes in characteristic silk fibroin secondary structure signals (such as β-sheet or random coil content) due to AST binding (37).

### 2.5 Stability tests of SFP-AST nanocomposites

#### 2.5.1 Effect of pH on stability

A “pH shock” experiment was conducted to evaluate the protective effect of SFP on AST under alkaline conditions. An SFP-AST nanocomposite solution (200 μL, containing AST at an initial pH of 4.0) was prepared as described above. Similarly, a control sample of free AST in aqueous solution (200 μL, pH 4.0) was prepared. Each sample was rapidly treated by adding an equal volume (200 μL) of 0.1 M NaOH, abruptly raising the pH. The mixture was briefly agitated, and changes in the UV-vis absorption spectrum were immediately recorded, focusing on the AST absorbance peak (~470 nm). The pH of the solution was monitored throughout the process. The absorbance values before and after NaOH addition were used to calculate the percentage of AST retained. All measurements were performed in triplicate. By comparing the AST retention in the presence and absence of SFP, we assessed how effectively the SFP matrix buffers the sudden increase in pH and protects AST from alkali-induced degradation.

#### 2.5.2 Storage stability

The stability of astaxanthin during storage in the nanocomposite was examined under ambient conditions. Samples (1 mL each) of SFP-AST nanocomposite and free AST solution (each containing the same initial concentration of AST) were placed in sealed 5 mL glass vials. The vials were stored at room temperature in the dark to prevent photo-degradation. At designated time intervals (0 h and 24 h), aliquots were taken and the UV-vis absorbance at ~470 nm was measured. The amount of AST remaining was quantified by comparing the absorbance at 24 h to the initial absorbance at 0 h (considering appropriate dilution factors if any). The percentage of AST retention after 24 h was calculated for both the free AST and SFP-AST samples. All experiments were performed in three independent replicates. A higher retention percentage in the SFP-AST sample, relative to free AST, would indicate that SFP confers improved storage stability to astaxanthin.

### 2.6 Antioxidant activity (DPPH radical scavenging assay)

Based on previous research, the free radical scavenging ability of AST was determined using the method reported, with minor modifications (38, 39). The antioxidant capacities of free astaxanthin and SFP - AST nanocomposites were evaluated using the DPPH (2,2-diphenyl-1-picrylhydrazyl) free radical scavenging assay (36). Briefly, a stock solution of DPPH (0.1 mM) was prepared by dissolving DPPH in absolute ethanol. In each assay, 0.4 mL of the 0.1 mM DPPH solution was mixed with 0.2 mL of the sample in a test tube (giving a final DPPH concentration of 0.067 mM). Three sample types were tested simultaneously: (1) SFP-AST nanocomposite solution, (2) free AST solution (at the same AST concentration as in the nanocomposite), and (3) SFP solution (at the same peptide concentration, without AST). Each mixture was briefly vortexed and then incubated at room temperature in the dark for 30 min. After incubation, the remaining DPPH concentration was measured by recording the absorbance at 517 nm using a UV-vis spectrophotometer. A control sample was prepared by mixing 0.4 mL of 0.1 mM DPPH solution with 0.2 mL of ethanol (without any sample) to obtain *A*_0_ (the absorbance of DPPH without any antioxidant). Additionally, for background correction, 0.2 mL of each sample was mixed with 0.4 mL of ethanol (without DPPH) to measure *A*_2_ (the absorbance of the sample itself, if any, at 517 nm). The DPPH scavenging percentage was then calculated using the equation:


$$\text{DPPH clearance}(\%)=(1-\frac{A_{1}-A_{2}}{A_{0}})\times 100\%$$

where *A*_0_ is the absorbance of the DPPH–sample mixture and *A*_1_ is the absorbance of the sample without DPPH (to account for any intrinsic absorbance of the sample). In this formula, (*A*_1_ – *A*_2_) represents the absorbance of DPPH remaining after reaction with the sample. All experiments were carried out in triplicate, and the results are reported as mean ± standard deviation. A higher DPPH scavenging percentage indicates greater antioxidant activity.

### 2.7 Statistical analysis

All experiments were performed in triplicate unless otherwise indicated. Data are expressed as mean ± standard deviation (SD). Statistical analyses were carried out using SPSS v26.0 (IBM Corp., United States). Independent-sample *t*-tests were applied for pairwise comparisons (e.g., free AST vs. SFP-AST). When more than two groups were compared, one-way analysis of variance (ANOVA) followed by Tukey’s post-hoc test was performed. Significance was accepted at *p* < 0.05 (*), *p* < 0.01 (**). Error bars shown in all figures represent SD.

## 3 Results and discussion

### 3.1 Interaction between astaxanthin and silk fibroin peptide

**Figure 1:**
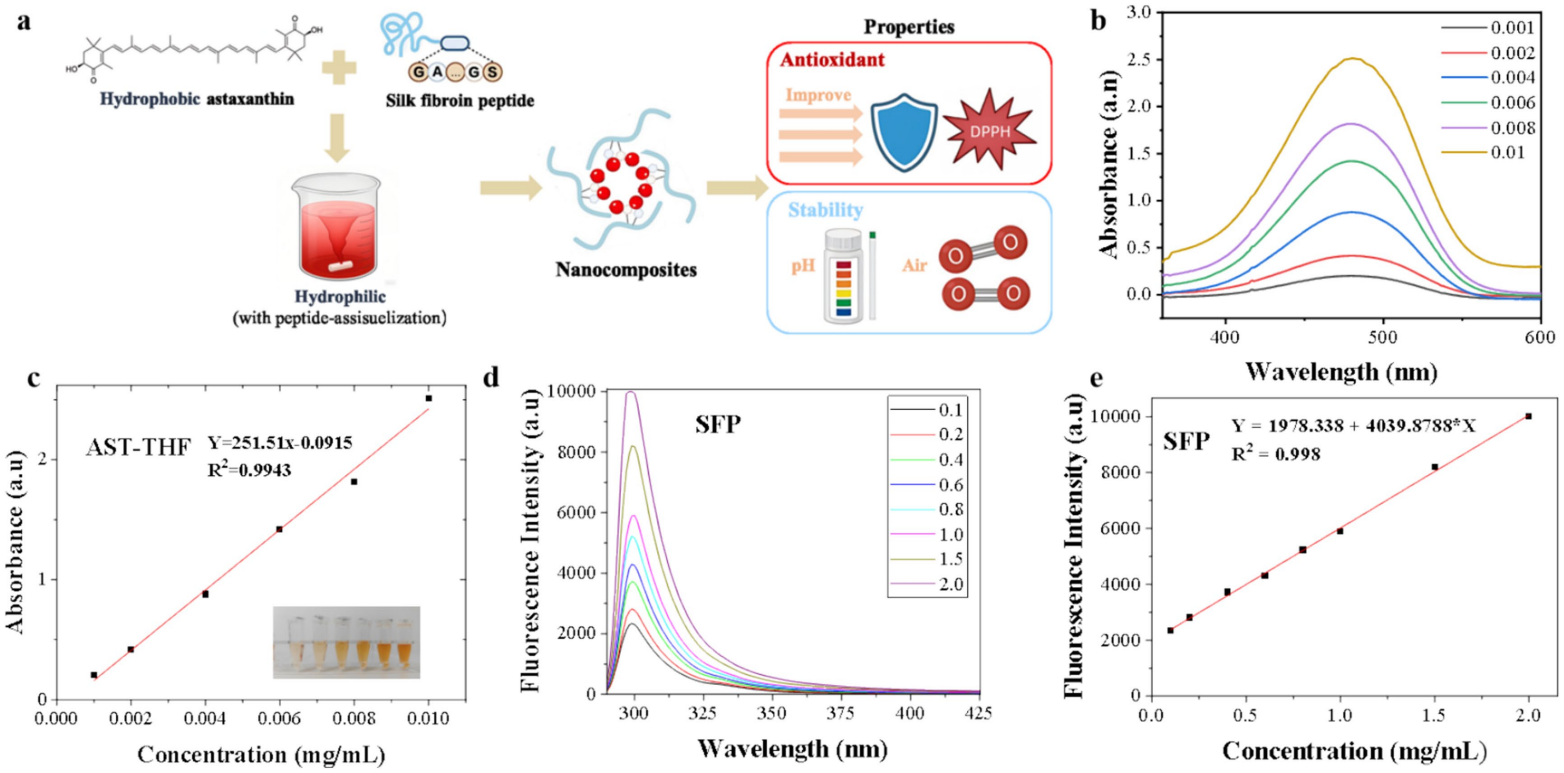
**(a)** Schematic diagram of the complexation process of silk fibroin peptide (SFP) with AST. **(b)** UV-Vis absorption results of different concentrations of astaxanthin (AST). **(c)** Standard curve of UV-Vis absorption of astaxanthin. **(d)** Fluorescence spectra results of different concentrations of SFP. **(e)** Standard curve of fluorescence spectra of different concentrations of SFP.

UV-vis absorption and fluorescence analysis: AST is classified as a keto-carotenoid (a terpenoid carotenoid with terminal keto groups) characterized by a conjugated polyene chain structure. In solution, astaxanthin exhibits a distinctive visible absorption peak around 470 nm. As shown in Figure 1b, the absorbance at 470 nm increases proportionally with AST concentration (the corresponding calibration curve is given in Figure 1c). UV-vis can be used to assess whether AST has been encapsulated by SFP to form nanoparticles (40). In our nanoprecipitation-based approach (Figure 1a, schematic), AST in THF was mixed with an aqueous SFP solution under stirring, leading to the formation of SFP-AST nanocomposites upon solvent evaporation. To identify an optimal SFP concentration for complexation, we first examined the intrinsic fluorescence of SFP at various concentrations and its quenching behavior upon AST addition. Figure 1d shows the fluorescence emission spectra of SFP at different concentrations, and Figure 1e presents the fluorescence intensity standard curve. Based on these preliminary tests, we selected an SFP concentration of 1 mg/mL for preparing the nano-precipitates, as this concentration provided a robust fluorescence signal and a pronounced quenching effect when AST was introduced (36).

**Figure 2:**
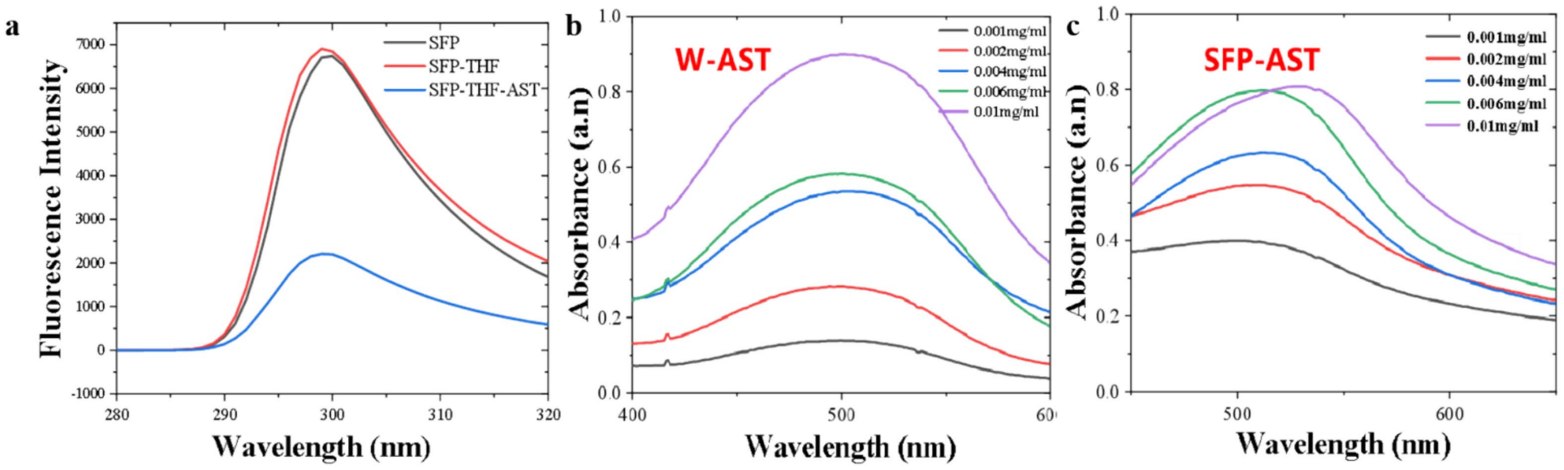
**(a)** Fluorescence burst of SFP with THF and AST **(b)** Peak UV absorption of aqueous solution of AST at different concentrations **(c)** Peak UV absorption of aqueous solution of SFP-AST at different concentrations (UV-Vis absorption spectra of SFP-AST nanocomposites at different AST concentrations).

Upon mixing AST with the SFP solution, a clear fluorescence quenching phenomenon was observed. Figure 2a illustrates that the fluorescence emission peak of SFP (centered around ~300 nm, due to aromatic amino acid residues in the peptide) was drastically reduced in intensity after AST was added, indicating that AST effectively quenched the intrinsic fluorescence of SFP (41). Fluorescence quenching occurs when the excited-state energy of a fluorescent molecule is dissipated through interactions with a quencher. In protein–ligand systems, quenching can result from hydrophobic interactions, electrostatic forces, van der Waals contacts, or hydrogen bonding between the protein (or peptide) and the ligand (42, 43). Such interactions can bring the quencher (AST, in this case) into proximity with fluorescent chromophores (e.g., tryptophan or tyrosine in SFP) or induce conformational changes, leading to a decrease in fluorescence intensity or a shift in the emission peak. Our results suggest that astaxanthin’s polyene cyclic structure interacts with hydrophobic regions of SFP, causing the observed fluorescence quenching. This indicates the formation of a binding complex between AST and SFP in solution.

We further used UV-vis spectroscopy to monitor the incorporation of AST into SFP nanoparticles. Figure 2b shows the UV-vis absorption spectra of astaxanthin solutions (in aqueous medium) after undergoing the same stirring and evaporation process used to create the nanocomposites (but without SFP), whereas Figure 2c shows the spectra of the corresponding SFP-AST nanocomposite solutions. Comparing these spectra provides insight into astaxanthin’s state and stability with and without the SFP matrix. In the AST-only samples (Figure 2b), the characteristic AST absorbance peak (initially ~470 nm, as in Figure 1b) remains in the vicinity of 470–500 nm after the processing step, though the intensity diminishes as the initial AST concentration is lowered. In contrast, the SFP-AST nanocomposites (Figure 2c) exhibit notable spectral shifts. The AST absorbance peaks in the presence of SFP are broadened and red-shifted, appearing in the 500–600 nm range, and their overall intensity is altered relative to the AST-only case. The appearance of these red-shifted absorption bands suggests the formation of AST-SFP assemblies or nanoparticles, where AST molecules may experience a different microenvironment (e.g., aggregated or bound state within SFP matrices) that affects their electronic absorption characteristics.

The UV-vis spectrum of blank SFP nanoparticles exhibited only a weak background signal in the 400–600 nm range and showed no characteristic absorption peak at 470 nm (Supplementary Figure S1). After subtraction of the blank nanoparticle background, the characteristic absorption band of AST showed reduced intensity and slight spectral changes in the SFP-AST system, suggesting that AST associated with SFP particles did not exhibit the same spectral behavior as free dissolved AST. Similar spectral changes following encapsulation have been reported previously, where interactions between astaxanthin and protein- or nanoparticle-based carrier matrices altered or shielded the conjugated polyene chromophore of AST (44). Furthermore, studies have shown that aggregation of free astaxanthin in aqueous systems is typically accompanied by pronounced spectral broadening and significant bathochromic or hypsochromic shifts (45). In the present study, such broadened or strongly shifted absorption bands were not observed in either the W-AST or SFP-AST samples, indicating that large free AST aggregates were unlikely to be present in the aqueous phase. Based on these observations, the absorbance measured at 470 nm was attributed primarily to free dissolved AST, and its concentration was estimated using a calibration curve constructed from free AST standards. The reduction in the apparent free AST concentration relative to the initial AST content was considered to represent the fraction of AST associated with SFP particles through adsorption and/or incorporation within the nanoparticle structure. The development of new absorbance features at longer wavelengths is a clear indication that SFP has successfully associated with AST to form nanocomposites (32).

Interestingly, the extent of astaxanthin retention after the nanoprecipitation process depended on the initial AST concentration. When the initial AST concentration was highest (0.01 mg/mL), the absorbance of the AST in the SFP-AST solution was actually lower than that of the corresponding AST-only solution at the same nominal concentration. This counter-intuitive result can be explained by a high encapsulation efficiency at 0.01 mg/mL AST: a significant portion of AST became bound within or shielded by SFP nanoparticles (perhaps making the solution more transparent to light), thus yielding a lower measured absorbance in the supernatant. In contrast, at lower initial AST concentrations (0.006 mg/mL and below), the SFP-AST samples showed higher absorbance than the free AST samples. In these cases, SFP was unable to encapsulate all of the astaxanthin; however, even partial association of AST with SFP appeared to improve AST’s stability against evaporation and oxidation, resulting in higher remaining AST levels compared to the unprotected control. In other words, astaxanthin that was combined with SFP suffered less loss (due to degradation or adsorption to the container) than astaxanthin alone, which is reflected in the relatively higher absorbance of the SFP-AST samples after processing. The UV-vis spectrum of blank SFP nanoparticles shows only a weak, background in the 400–600 nm region, with no absorption peak at 470 nm (Supplementary Figure S1). As is apparent, the absorption peak is unaffected by free SFP particles. Among all the tested concentrations, an initial AST concentration of 0.01 mg/mL yielded the greatest AST retention following the stirring/evaporation treatment. Therefore, we chose 0.01 mg/mL as the working AST concentration for subsequent characterization, stability, and antioxidant experiments. Based on the absorbance measurements before and after nanocomposite formation, the encapsulation efficiency of AST by SFP at this condition was calculated to be approximately 6%. Although this encapsulation efficiency is relatively modest, the improvements in AST’s stability and functionality (detailed below) demonstrate the effectiveness of the SFP matrix even at low loading (16). The relatively low encapsulation efficiency (~6%) observed in this study may be attributed to the low molecular weight of SFP and the rapid nanoprecipitation process, which could limit the formation of stable encapsulating structures. Additionally, the strong hydrophobicity and low concentration of AST may hinder its effective incorporation. Nevertheless, the significant enhancement in stability and antioxidant activity suggests that even partial association with SFP is sufficient to improve AST performance. Future studies may focus on optimizing SFP/AST ratio, nanoprecipitation conditions, and peptide molecular structure to improve encapsulation efficiency. The SFP-AST system in this study represents a co-assembled nanocomposite in which bound and free AST may coexist. Therefore, all characterizations were conducted on the whole system to reflect its overall functional performance.

### 3.2 Characterization of SFP-AST nanocomposites

To confirm the formation of nano-complexes and to understand their structural features, we characterized the SFP-AST nanocomposites using TEM, DLS, CD, and FTIR techniques. These analyses provide complementary information on particle morphology, size distribution, and the molecular interaction between SFP and AST.

**Figure 3:**
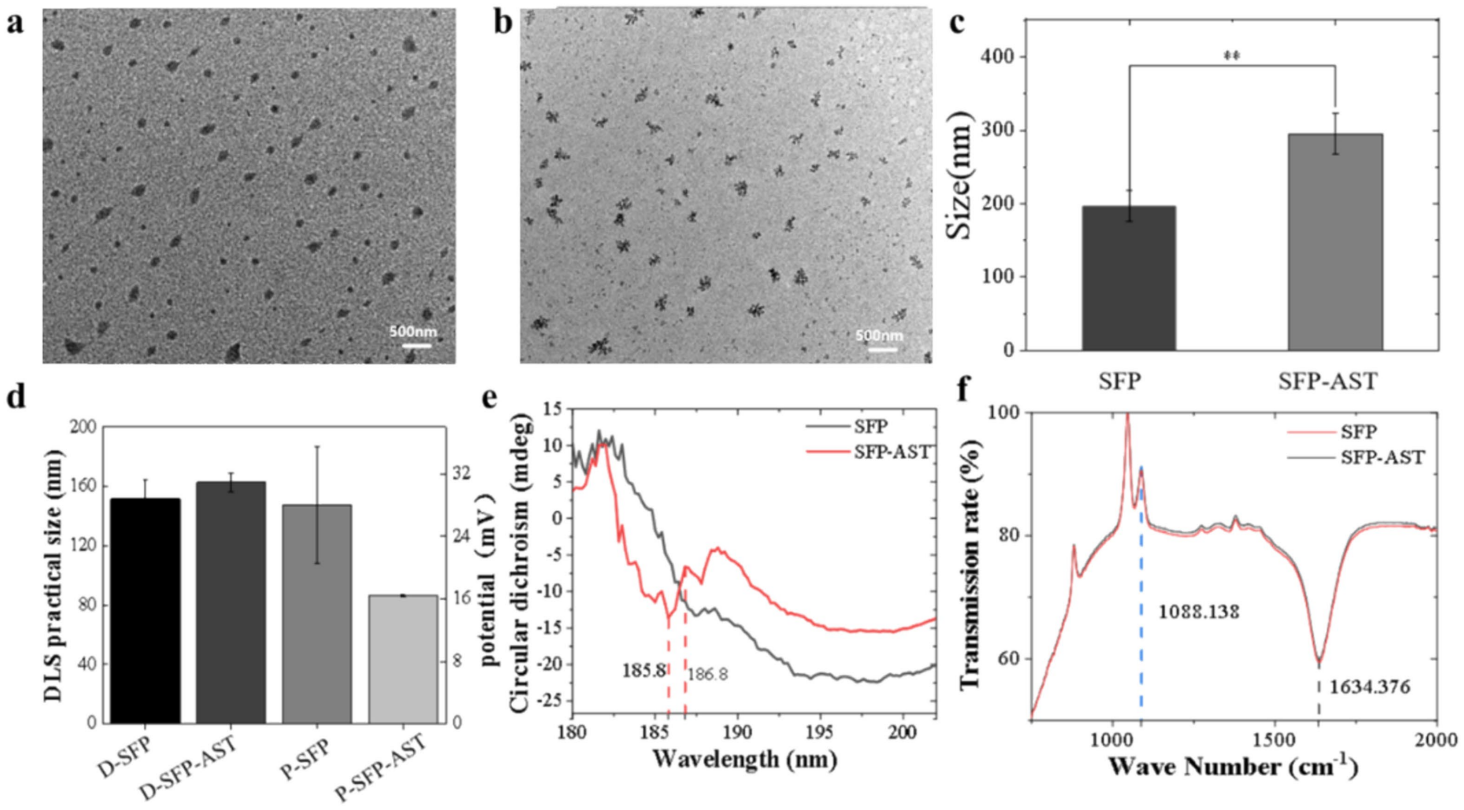
**(a)** Transmission electron microscopy (TEM) characterization of SFP. **(b)** Transmission electron microscopy (TEM) characterization of SFP-AST nanocomposites. **(c)** Mean statistical particle size of SFP and SFP-AST nanocomposites. **(d)** Hydrodynamic diameter and zeta potential statistics of SFP and SFP-AST nanocomposites. **(e)** Circular dichroism (CD) spectra of SFP and SFP-AST nanocomposites. **(f)** Fourier transform infrared (FTIR) spectra of SFP and SFP-AST nanocomposites; **p ≤ 0.01.

*Morphology and size (TEM & DLS)*: The TEM micrographs in Figures 3a–c reveal distinct differences between SFP alone and the SFP-AST composite. Silk fibroin peptide alone (Figure 3a) was observed to self-assemble into roughly spherical nano-sized particles with an average diameter of about 196.9 ± 21.4 nm. These SFP nanoparticles likely result from the hydrophobic segments of the peptides aggregating in aqueous solution. After binding with astaxanthin, the morphology of the particles changed noticeably. The SFP-AST sample (Figure 3b) showed irregularly shaped nanoparticles, and the average particle size increased to approximately 295 ± 28.0 nm. The formation of larger, less regular particles upon AST addition is evidence of successful incorporation of AST into the SFP assemblies. The presence of astaxanthin could promote the clustering of SFP particles or induce a different assembly structure due to hydrophobic interactions, leading to the observed size increase (29). A statistical comparison of particle size distributions (Figure 3c) confirms the size increase from SFP to SFP-AST nanoparticles, corroborating that AST has bound to SFP and altered its assembly behavior.

Dynamic light scattering measurements further quantified the hydrodynamic size and surface charge of the nanocomposites. The DLS results (Figure 3d) showed that SFP alone had an average hydrodynamic diameter of about 151.8 ± 12.5 nm with a positive ζ-potential of +28.0 ± 7.5 mV. The relatively high positive zeta potential of SFP particles can be attributed to the protonation of amino groups in the peptide at the measurement pH, which confers colloidal stability via electrostatic repulsion. Upon forming SFP-AST nanocomposites, the hydrodynamic diameter was measured to be 162.7 ± 6.5 nm, and the ζ-potential decreased to +16.4 ± 0.2 mV. The slight increase in DLS size for SFP-AST is consistent with the TEM observations of larger composite particles. This may be due to the increased hydrophilicity of the outer layer of the SFP-AST nanocomposite, which causes an increase in the hydrodynamic diameter measured by dynamic light scattering (46). The reduction in zeta potential suggests that some of the positive charges on SFP may be neutralized or shielded by the bound astaxanthin molecules, or by subtle changes in peptide conformation and surface chemistry after binding AST. Nonetheless, the SFP-AST nanocomposites maintained a positive surface charge and a fairly narrow size distribution, indicating they were still well-dispersed and colloidally stable in aqueous solution. The formation of these stable SFP-AST nanoparticles in water is a key outcome, as it enables astaxanthin—normally hydrophobic and water-insoluble—to exist in an aqueous, biologically relevant environment with improved stability. It is important to note that the particle size values displayed in Figure 3c correspond to the mean hydrodynamic diameters, while Figure 3d presents the size distribution profiles derived from DLS measurements. It is important to note that discrepancies may be perceived between these representations due to variations in distribution shape and peak width. Furthermore, slight variations are to be expected due to the dynamic nature of the system and measurement sensitivity.

*Secondary structure analysis (CD & FTIR)*: To investigate whether the binding of astaxanthin affected the secondary structure of the silk fibroin peptide, we analyzed the SFP-AST nanocomposites using circular dichroism and FTIR spectroscopy. The CD is a sensitive method for monitoring protein secondary structure or conformational changes during the binding process of protein and ligand (47). The far-UV CD spectra (Figure 3e) of SFP before and after AST addition were essentially overlapping, indicating no major change in the peptide’s secondary structure content. The CD spectrum of SFP exhibited a characteristic negative ellipticity peak at ~185.8 nm and a positive peak at ~196.8 nm. (These two closely spaced peaks may represent a split α-helix or β-sheet signal, or a fine-structure splitting of the π → π* transition in the silk fibroin peptide, which contains a mixture of random coil and β-sheet conformations.) Importantly, the SFP-AST composite showed the same spectral features at these wavelengths with nearly identical intensity, suggesting that the peptide’s conformation remained unchanged upon forming the nanocomposite with AST (48).

The FTIR analysis corroborated the CD findings. Figure 3f displays the ATR-FTIR spectra focusing on the amide I region (1600–1700 cm^−1^), which is sensitive to protein secondary structure. The silk fibroin peptide alone showed an amide I band with a prominent component at around 1,634 cm^−1^, which is indicative of β-sheet structure (a known feature of silk fibroin-derived peptides) (42). In the SFP-AST nanocomposite spectrum, the amide I band profile was almost unchanged: the peak at ~1,634 cm^−1^ remained at the same position and with nearly the same intensity as in the free SFP spectrum, and no new peaks or significant shifts were observed. The overlapping of the amide I bands confirms that the secondary structure (particularly the β-sheet content) of SFP was not disrupted by the binding of astaxanthin. At the same time, the absence of new characteristic peaks in the spectrum indicates that no new covalent bonds were formed during the complexation process, suggesting that the system primarily relies on noncovalent interactions (such as hydrogen bonding, hydrophobic interactions, and possible π–π interactions) to maintain structural stability. This finding is consistent with results from related studies on protein hydrolysate delivery systems, which indicate that structural rearrangement and intermolecular interactions are key factors in stabilizing active substances. In summary, the formation of the SFP-AST complex is primarily driven by various noncovalent interactions, which is also a key factor contributing to its enhanced stability and functionality.

Together, the CD and FTIR results demonstrate that SFP acts as a relatively “rigid” host matrix that binds AST through surface interactions rather than through a co-assembly mechanism that would involve substantial conformational rearrangement of the peptide. We infer that the interaction between SFP and AST is primarily driven by non-covalent molecular forces (hydrophobic attractions being most likely, given astaxanthin’s hydrophobic nature), and that AST binds to pre-existing hydrophobic sites or pockets on the SFP nanoparticles. This binding mode is markedly different from what has been reported for some other peptide–pigment systems. For example, demonstrated a co-assembly approach where an amphiphilic peptide was specifically designed to interact with the anthocyanin cyanidin-3-O-glucoside (C3G). In that system, the peptide (which contained hydrophobic amino acids like tryptophan) underwent a conformational change upon binding C3G, forming a new secondary structure that encapsulated the anthocyanin in a core-shell nanostructure. In our SFP-AST system, however, the absence of any significant secondary structure change in SFP suggests that astaxanthin binding occurs by a surface association or intercalation into existing SFP particle structures, rather than by inducing a peptide folding/unfolding event. This distinction is important: it implies that SFP can stabilize AST by simple binding and shielding, without the need for complex structural transformations, which could be advantageous for preserving the functional integrity of both the carrier and the active compound.

### 3.3 Stability of SFP-AST nanocomposites

Having confirmed the successful formation of SFP-AST nanocomposites, we next evaluated how the SFP matrix influences the stability of astaxanthin under stressful conditions. In particular, we investigated AST stability against sudden exposure to high pH (alkaline conditions) and during short-term storage at room temperature. We hypothesized that complexation with SFP would protect astaxanthin from degradation in these conditions by providing a microenvironment that buffers external changes and possibly by physically shielding AST from reactive species (e.g., OH^−^ or oxygen).

#### 3.3.1 Effect of pH on AST stability

**Figure 4:**
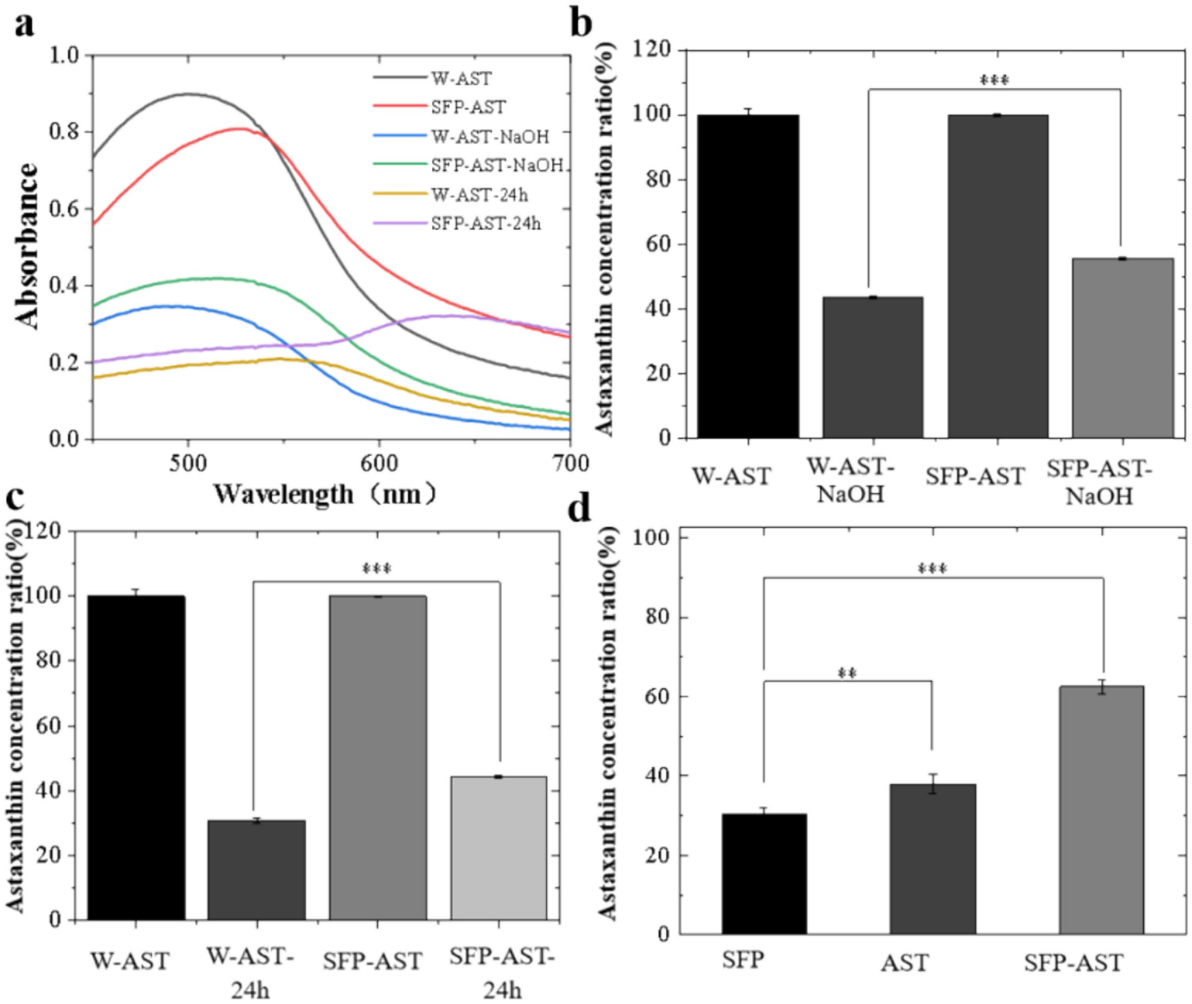
**(a)** Stability of AST and SFP-AST nanocomposites under the influence of NaOH and during storage in air **(b)** Analysis of significant differences in the stability of AST and SFP-AST nanocomposites under the influence of NaOH. **(c)** Analysis of significant differences in the storage stability of AST and SFP-AST nanocomposites **(d)** DPPH scavenging rate of AST and SFP-AST nanocomposites; **p ≤ 0.01; ***p ≤ 0.001.

Astaxanthin is known to be unstable in strongly basic environments, typically undergoing structural degradation or isomerization when the pH is raised. To test the protective effect of SFP, we subjected both free AST and SFP-AST samples to an abrupt pH increase and monitored the loss of astaxanthin. Prior to NaOH addition, both samples had an acidic pH (around 4.0) and exhibited their characteristic absorption (~470–500 nm). Upon adding 0.1 M NaOH, the pH of each sample rapidly shifted to alkaline (pH > 12). This led to immediate spectral changes (Figure 4a). In the case of the free astaxanthin solution (denoted W-AST for “astaxanthin in water”), the main AST absorbance peak dramatically decreased in intensity and also underwent a slight red shift. Quantitatively, the peak absorbance of the free AST dropped from 0.899 (at pH 4.0) to 0.345 after NaOH addition. This corresponds to only 43.6% AST retention (relative to the initial absorbance). Visually, the AST solution faded in color, consistent with substantial degradation. In contrast, the SFP-AST nanocomposite showed a more moderate decrease: its absorbance peak decreased from 0.808 to 0.420 under the same pH shock, corresponding to 55.6% AST retention. Moreover, the SFP-AST sample’s absorbance peak exhibited a more pronounced red shift than the free AST (shifting deeper into the 500+ nm range), which could indicate some changes in AST’s microenvironment or aggregation state with SFP under high pH. The higher residual absorbance and AST content in the SFP-AST sample demonstrates that SFP provided a degree of protection to astaxanthin against the alkali. We postulate that the silk peptide, with its amphoteric nature, acted as a pH buffer and a physical stabilizer: the SFP could locally neutralize some of the added OH^−^ and/or shield AST from direct exposure, thus slowing the degradation of AST. Statistical analysis of multiple trials (Figure 4b) confirmed that the astaxanthin retention in SFP-AST was significantly greater than in the free AST solution after the pH jump (*p* < 0.05). These results support the conclusion that incorporating astaxanthin into SFP nanoparticles enhances its resistance to alkaline degradation.

It should be noted that the buffering effect of SFP proposed in this study is inferred indirectly from stability results. Direct measurement of pH changes was not performed. Future work will focus on monitoring dynamic pH variations to better understand the potential buffering behavior and underlying stabilization mechanisms.

#### 3.3.2 Storage stability at ambient conditions

We further compared the stability of astaxanthin in free versus SFP-bound form during storage over 24 h at room temperature (in the dark). Figure 4c summarizes the changes in AST absorbance for both samples over the storage period. The free AST solution showed a substantial decline in absorbance at 470 nm, dropping from an initial value of 0.899 to 0.210 after 24 h. This indicates that only about 30.8% of astaxanthin remained in the free solution (a loss of ~69.2% in 1 day), highlighting astaxanthin’s poor stability even under mild storage conditions. In contrast, the SFP-AST nanocomposite retained a much higher fraction of astaxanthin. Its absorbance went from 0.808 initially to 0.320 after 24 h. Taking into account the slightly lower starting absorbance, this corresponds to approximately 44.4% of astaxanthin remaining after 24 h in the nanocomposite (~55.6% loss). In other words, the SFP-AST system maintains higher levels of AST than the free solution does over the same time period. The improved retention of AST in the presence of SFP can be attributed to several factors: the SFP peptide may hinder oxidative degradation of AST by restricting its exposure to oxygen or other reactive species, and the physical encapsulation/association might reduce the tendency of AST to aggregate or adsorb to container surfaces (common pathways for apparent loss of hydrophobic compounds in solution). The data clearly show that SFP incorporation significantly slows down the degradation of astaxanthin during storage. This suggests that the system has potential for applications requiring short-term stabilization, while further optimization is needed for long-term or more demanding conditions. The differences between the two samples were statistically significant (*p* < 0.05), as indicated by the analysis in Figure 4c. These findings suggest that SFP is a promising natural stabilizer capable of maintaining the potency of astaxanthin over the short term, which is particularly valuable for the development of functional foods or supplements rich in astaxanthin with a longer shelf life.

### 3.4 Antioxidant activity of SFP-AST nanocomposites

Astaxanthin’s strong antioxidant activity is one of its most important functional properties. After incorporating AST into the SFP nanocomposite, it was critical to verify whether AST’s antioxidant capacity was retained or even enhanced. We used the DPPH free radical scavenging assay to compare the antioxidant efficacy of free AST versus SFP-bound AST. The results are presented in Figure 4d. Free astaxanthin in ethanol (at the tested concentration) exhibited a DPPH radical scavenging rate of 38.11% ± 1.71%, reflecting AST’s ability to donate electrons/hydrogen atoms to neutralize DPPH radicals. In comparison, the SFP-AST nanocomposite achieved a significantly higher scavenging rate of 62.40% ± 1.63%under the same conditions. This ~1.6-fold increase in DPPH scavenging suggests that the nanocomposite formulation not only preserved AST’s antioxidant function but actually improved its efficacy. The enhancement could be due to better dispersion of AST molecules in the aqueous phase when bound to SFP, leading to more accessible interaction with DPPH radicals. The antioxidant activity of the SFP-AST system is attributed to the combined contributions of both SFP and AST, indicating a combined effect. While silk fibroin peptide is not a strong antioxidant itself, it did show a small DPPH scavenging effect (30.18% ± 1.71% in the SFP-only sample, likely due to residual amino acids with weak radical-scavenging ability). Regardless, the antioxidant activity of AST was clearly maintained in the nanocomposite, indicating that the binding to SFP did not quench AST’s redox-active sites. On the contrary, the nanocomposite’s higher activity suggests that astaxanthin is more effectively presented to the radicals or stabilized in an active form. The differences between free AST and SFP-AST in this assay were statistically significant (*p* < 0.01). These results demonstrate that forming nanocomposites with SFP does not impede the biological function of astaxanthin; rather, it can amplify its beneficial antioxidant effect, which is advantageous for any application aiming to harness astaxanthin’s bioactivity (15).

## 4 Conclusion

In this study, we successfully prepared silk fibroin peptide-astaxanthin (SFP-AST) nanocomposites using the nanoprecipitation method. The analysis of the particle size distribution indicated that the nanocomposites exhibited a particle size range of 150 to 300 nm, thereby suggesting that the encapsulation of astaxanthin by the silk peptides had been accomplished, resulting in the formation of a stable nanostructure. The Fourier-transform infrared (FTIR) analysis indicated that the distribution of the Amide I band remained virtually unchanged, suggesting that the secondary structure of SFP was not disrupted by AST. The results suggest that the formation of the composite is primarily driven by non-covalent interactions, such as hydrogen bonding and hydrophobic interactions. This is similar to protein hydrolysate carrier systems reported in the literature (e.g., collagen hydrolysate), which stabilize active substances through intermolecular interactions. In the course of stability tests, the SFP-AST nanocomposite demonstrated superior stability in comparison with free astaxanthin under conditions of alkaline shock and storage. It is noteworthy that under conditions of alkaline shock, the composite exhibited a significantly higher astaxanthin retention rate of 55.6%, in comparison to the 43.6% retention rate observed for free astaxanthin. Furthermore, following a 24-h storage period, the astaxanthin retention rate of the SFP-AST nanocomposite was found to be 44.4%, which is significantly higher than the 30.8% observed for free astaxanthin. This finding suggests that SFP effectively enhances the stability of astaxanthin. With regard to antioxidant activity, the DPPH radical scavenging rate of the SFP-AST nanocomposite was 62.40%, which is significantly higher than the 38.11% observed for free astaxanthin. This finding indicates that the antioxidant performance of the nanocomposite has been enhanced. In summary, this study successfully developed a novel SFP-AST nanocomposite that significantly improves the stability and antioxidant activity of astaxanthin. The findings of this study suggest that silk fibroin peptide is an effective biocompatible carrier capable of improving the stability and bioactivity of hydrophobic bioactive substances. Future studies should further optimize the ratio of silk fibroin peptide to astaxanthin in order to improve encapsulation efficiency and investigate the long-term stability of the composite under various environmental conditions. In addition, further *in vivo* experimentation is required to evaluate the biological activity of the SFP-AST nanocomposite, with a view to further validating its potential applications in functional foods, pharmaceuticals and cosmetics. Concurrently, the investigation of the biodegradability and toxicity of the nanocomposite is imperative for the comprehensive evaluation of its safety. Despite the fact that this study has yielded favorable results, the encapsulation efficiency of the nanocomposite still requires enhancement and can be further optimized through the adjustment of processing parameters in future research.

## Data Availability

The original contributions presented in the study are included in the article/Supplementary material, further inquiries can be directed to the corresponding author.
