# Supplementary material for: Fabrication of silk fibroin peptide–astaxanthin nanocomposites by nanoprecipitation for enhanced stability and antioxidant activity
Source: Front Nutr. 2026 Jul 7;13:1803229. doi: 10.3389/fnut.2026.1803229 (PMC13385685; doi:10.3389/fnut.2026.1803229)
Supplement: Supplementary file 1 [file Table_1.docx]

**Supplimentary information for:**

**Fabrication of Silk Fibroin Peptide–Astaxanthin Nanocomposites by Nanoprecipitation for Enhanced Stability and Antioxidant Activity**

Stopira Yannick Benz Boboua^a,b,1^, ShaoPeng Chen^b,1^, Yong Sun^a^, Mengjie Li ^c^, Ying Guo^b^, QingMei Wen^b^, Yilu Chen^d^, Zeng Fan^f^, Tao Zheng^b,*^

^a^College of Engineering, Northeast Agriculture University, Harbin 150030, PR China.

^b^Guangzhou Institute of Energy Conversion, Chinese Academy of Sciences, Guangzhou, 510640, China

^c^College of Grassland Science and Technology, China Agricultural University, Beijing 100091, China

^d^Ecogreen Biotechnology Research Institute, Nanjing, 211100, China

^f^School of Environmental Engineering, Nanjing Institute of Technology, Nanjing, Jiangsu, China, CN211167

* Corresponding author:

Tao Zheng,

Guangzhou Institute of Energy Conversion, Chinese Academy of Sciences,Guangzhou 510640, P.R. China.

E-mail: [zhengtao@ms.giec.ac.cn](mailto:zhengtao@ms.giec.ac.cn)

Number of pages:2

Number of figures:1

**

Figure S1. **UV absorption peak of the SFP aqueous solution.**
